# Comprehensiveness cuproptosis related genes study for prognosis and medication sensitiveness across cancers, and validation in prostate cancer

**DOI:** 10.1038/s41598-024-57303-8

**Published:** 2024-04-26

**Authors:** Longfei Yang, Yifan Tang, Yuwei Zhang, Yang Wang, Peng Jiang, Fengping Liu, Ninghan Feng

**Affiliations:** 1 Medical School of Nantong University, Nantong, 226001 China; 2 Department of Urology, Jiangnan University Medical Center, 68 Zhongshan Road, Wuxi, 214002 Jiangsu China; 3 Wuxi School of Medicine, Jiangnan University, Wuxi, China; 4 Department of Urology, Wuxi No. 2 Hospital, Nantong University, Wuxi, China; 5 Department of Urology, Wuxi No. 2 Hospital, Nanjing Medical University, Wuxi, China

**Keywords:** Pan-cancer analysis, Cuproptosis-related genes, Prognosis, Tumor microenvironment, Drug sensitivity, Cancer, Computational biology and bioinformatics, Oncology

## Abstract

Cuproptosis-related genes (CRGs) are important for tumor development. However, the functions of CRGs across cancers remain obscure. We performed a pan-cancer investigation to reveal the roles of CRGs across cancers. In an analysis of 26 cancers, 12 CRGs were differentially expressed, and those CRGs were found to have prognostic value across different cancer types. The expression of CRGs exhibited varied among tumors of 6 immune subtypes and were significantly correlated with the 16 sensitivities of drugs. The expression of CRGs were highly correlated with immunological subtype and tumor microenvironment (TME) of prostate cancer. We also established CRGs-related prognostic signatures that closely correlated with prognosis and drug sensitivity of prostate cancer patients. Single-cell RNA-seq revealed that several CRGs were enriched in the cancer cells. Finally, an in vitro experiment showed that elesclomol, a cuproptosis inducer, targets ferredoxin 1 and suppress cell viability in prostate cancer cells. In conclusion, we carried out a comprehensive investigation for determining CRGs in differential expression, prognosis, immunological subtype, TME, and cancer treatment sensitivity across 26 malignancies; and validated the results in prostate cancer. Our research improves pan-cancer knowledge of CRGs and identifies more effective immunotherapy strategies.

## Introduction

Recently, there has been a significant rise in cancer prevalence and fatality rates, highlighting that it poses a severe threat to global health and quality of life^1^. Tumor immunotherapy has become an attractive strategy for treating cancer, with a particular focus on targeting programmed cell death-1 (PD-1)/programmed cell death-ligand 1 (PD-L1) or cytotoxic T lymphocyte-associated antigen-4 (CTLA4)^2^. Nevertheless, cancer cells have evolved intricate mechanisms to elude immune system responses. For example, mutations in beta 2-microglobulin (β2MG) can lead to the deletion of human leukocyte antigen (HLA) molecules, hence causing a lack of neoantigens expressed on the cellular surface. Due to toxic T-cell dysfunction, PD-1 drug resistance occurs in patients^3^. In addition, the financial implications of cancer treatment place a significant economic strain on families and societies worldwide^4^. There is a pressing imperative to advance discovery of novel biomarkers for diagnosis and targets for treatment of cancer.

As a vitally important mineral for human health, copper plays a crucial function in a variety of pathological and physiological processes^5,6^. Recent investigations have demonstrated that copper concentrations are notably increased in plasma and cancerous tissues of cancer patients versus healthy individuals^7^. Another research showed that cancer development and progression might be affected by changes in intracellular copper levels^8^. In fact, several studies have indicated that copper chelators (tetrathiomolybdate) and copper ionophores (disulfiram) can be used in cancer therapy^9,10^. Nevertheless, even minimal intracellular levels of copper might trigger cellular demise^11^. Interestingly, cuprotosis, is induced by elesclomol (ES), a novel mechanism of cellular demise, which direct binding affinity towards lipoylated constituents of tricarboxylic acid cycle, hence inducing cellular demise^12^. In other words, targeting cuprotosis-related genes (CRGs) may be a potential strategy for avoiding copper toxicity and developing a novel cancer therapeutic target. However, there are few findings about CRGs and 26 malignancies. As a result, we analyzed CRGs across various malignancies.

In this study, the RNA sequence expression data of 26 tumors, clinical information, stemness score data, immunological subtype data of CRGs, and gene expression data of 26 kinds of healthy tissues of CRGs were initially obtained from the UCSC Xena database. Multiple R packages were applied to conduct differential expression analysis and correlation analysis across tumors. To examine the associations between CRGs and survival outcomes across different forms of cancer, we used Cox regression and Kaplan–Meier analyses. Furthermore, association among the expression of CRGs, immunological types, tumor microenvironment (TME), stemness scores, and treatment sensitivity in distinct malignancies was calculated. On the basis of the CRGs, prostate cancer patients were classified into two clusters, and a variety of statistical methods were used to evaluate this gene pattern. The connection between the half-maximal inhibitory concentration (IC_50_) and risk scores of chemotherapeutic drugs was also evaluated using co-expression analysis. Integration of bulk RNA-seq samples was used to discover in greater detail the expression CRGs. Finally, a validation experiment was carried out on prostate cancer cells to confirm the above findings. The above comprehensive study paved the way for the identification of novel functions of CRGs and the development of novel chemotherapeutic approaches in the study across cancer.

## Materials and methods

### Patients and datasets

Gene expression profiles, survival data, the stemness scores and immune subtypes of 26 malignancies of The Cancer Genome Atlas (TCGA) database, and the RNA expression profiles of 26 kinds of normal tissues from the Genotype-Tissue Expression (GTEx) database, were extracted from UCSC Xena database (https://xenabrowser.net/datapages/). Next, we used “limma” package to combine the mRNA expression data of TCGA tissues and normal tissues of GTEx into a complete cohort after Log2 transformation. Supplementary Table S1 provides the abbreviations and complete names of 26 cancers.

### Correlation analysis and differential expression of CRGs in distinct cancer types

We obtained 12 CRGs, including solute carrier family 31 member 1 (SLC31A1), dihydrolipoamide *S*-acetyltransferase (DLAT), ATPase copper transporting beta (ATP7B), dihydrolipoamide dehydrogenase (DLD), dihydrolipoamide branched chain transacylase E2 (DBT), dihydrolipoamide *S*-succinyltransferase (DLST), glycine cleavage system protein H (GCSH), lipoic acid synthetase (LIAS), ferredoxin 1 (FDX1), lipoyltransferase 1 (LIPT1), pyruvate dehydrogenase E1 subunit beta (PDHB), and pyruvate dehydrogenase E1 subunit alpha 1 (PDHA1), based on the mechanical of copper induced cell death from the previous research^12^. To assess the expression of 12 CRGs across malignancies, by using “ggpubr” R package and displayed by boxplot.

### Analysis of the connection between CRGs expression and prognosis

The overall survival (OS) of TCGA patients was assessed using analysis of Kaplan–Meier (KM) and univariate Cox regression. Applying the R packages “survminer” and “survival”, the connection between survival and CRGs expression in diverse malignancies were evaluated.

### Analysis of the correlation between CRGs and immune subtypes across cancers

The “limma” package has been utilized to investigate the differences in CRGs expression in six immunological subtypes, including inflammatory, wound healing, transforming growth factor beta (TGF-β) dominant, immunologically quiet, lymphocyte-depleted, and interferon gamma (IFN-γ) dominant^13^. The above analysis was pictured by boxplot using “ggplot2” and “reshape2” R packages.

### Analysis of correlation between CRGs and tumor stemness scores and TME

The link between tumor stem cells and CRGs expression was examined using the R packages “corrplot” and “limma.” To assess the association between CRGs and the TME in various forms of malignancy, we utilized “estimate” and “corrplot” R packages.

### Analysis of correlation of CRGs and treatment sensitivity

CellMiner database was queried for the data of treatment sensitivity gene expression (https://discover.nci.nih.gov/cellminer/home.do). We conducted an assessment of the associations among drug sensitively and CRGs expression, employing the R packages “ggpubr,” “limma,” and “impute.”

### Analysis of the connection between CRGs expression and tumor immunity in prostate cancer

To examine the connection between CRGs expression and the TME in prostate cancer, we operated “estimate,” “corrplot,” and “limma” R packages. Using the “limma” package, we examined the expression of CRGs in six immunological subgroups to determine the connection between CRGs and prostate cancer.

### Establishing and verification of cuprotosis-related prognostic signature

All CRGs were integrated into analysis of least absolute shrinkage and selection operator (LASSO) for determining hub CRGs and obtaining a risk signature by utilizing “glmnet” package. The prostate cancer patients were divided into two categories, and risk scores were measured as follows: riskscore = Σexpgenei * βi. Risk model performance was assessed using analysis of KM and area under curve (AUC). Survival rates between high-risk (HR) and low-risk (LR) groups were compared using “survminer” and “survival” packages. The “pheatmap” package was used to plot survival status and risk score distribution.

### Therapeutic response prediction

IC_50_ values of anticancer medications were assessed using the “pRRophetic” R package, and an examination was conducted to determine the variations in sensitivity to anticancer drugs between the HR and LR groups.

### Acquiring and analyzing single-cell sequencing data

The Gene Expression Omnibus (GEO) database was employed to retrieve the single-cell (sc) RNA sequencing dataset GSE141445. Seurat objects are generated through the operation of “Seurat” R package, wherein cells of superior quality are integrated into subsequent analytical procedures. We omitted cells containing fewer than 50 genes; fewer than three genes, and more than 5% mitochondrial genes. All of the data were log normalized, and then the 1500 genes with the biggest coefficient of variation between cells were filtered out. To reduce the dimensions of scRNA-seq data, the t-distributed stochastic neighbor embedding (tSNE) algorithm was utilized. Furthermore, algorithm “FindAllMarkers” was utilized to obtain marker genes that were differentially expressed in distinct cell clusters. Following this, the cells were labeled using the marker genes and analyzed with the “SingleR” package to identify different cell subgroups.

### Cell culture and chemicals

Prostate cancer cell lines DU145, PC-3, and LNCaP and normal adult human prostatic epithelial cells RWPE-1 all supplied by Procell (Wuhan, China). All four cell lines were grown in 1640 complete medium. All of the cell types grew in an incubator at 37 °C with 5% CO_2_. Elesclomol (MCE, United States) was diluted to a 100 nmol/L concentration in a cell culture medium. R1881 (RHAWN Chemical Technology Co., Ltd Shanghai China) was diluted to a 0.1 nmol/L concentration in a cell culture medium.

### RNA extraction and real-time PCR assays

RNA reagent (Vazyme, Nanjing, China) was applied to extract RNA from the aforementioned cells. Real-time quantitative polymerase chain reaction (qRT-PCR) was then operated for further detection. The primers used in this research are listed in Supplementary Table S1. The 2^−ΔΔCT^ method was applied to measure the gene expression.

### Western blotting

To extract cell protein, a RIPA lysis buffer comprising a protease inhibitor cocktail was applied. After that, sodium dodecyl sulfate polyacrylamide gel electrophoresis was used to separate similar amounts of proteins in the cell lysates, and the proteins were then electrophoretically moved onto polyvinylidene fluoride membranes. Afterward, blocked for 1 h with 5% skim milk, the membranes were hatched with rabbit FDX1 antibody (#12592-1-AP) and mouse-actin antibody (#66009-1-Ig) for 13 h at 4 °C. On the subsequent day, the membrane was subjected to secondary antibodies for a duration of 1.5 h at room temperature. Images were visualized utilizing enhanced chemiluminescence.

### CCK-8 assay

The cells were transferred to 96-well plates at a final concentration of 3000 cells in 100 μL in triplicate after 16 h of ES treatment and incubated in a humid incubator. The absorbance was assessed by applying microplate reader after a 30-min incubation with CCK-8 reagent (Vazyme Biotech).

### Caspase3/7 activity assay

Cells were rinsed twice with a new, warm medium, as well as caspase3/7 activity solution was added to each well (Beijing BioRab Technology Co. Ltd., China). A microplate reader measured fluorescence intensity 30 min after incubation at 37 °C and 5% CO_2_ (Biotek Gen5).

### Cell cycle assay

After incubation with ES, cells were collected and washed by phosphate-buffered saline and subsequently treated with ice-cold 75% ethanol 14 h at − 20 °C. Cell cycle assay kit was (solarbio Bioscience and Technology Co., LTD, Beijing, China) used to analyze cell cycle distribution.

### Statistical analysis

GraphPad Prism version 8.0 and R version 4.3.1 were carried out for all statistical tests. The cutoff for statistical significance was set at *P* < 0.05.

## Results

### Differential CRGs expression across cancers

**Figure 1:**
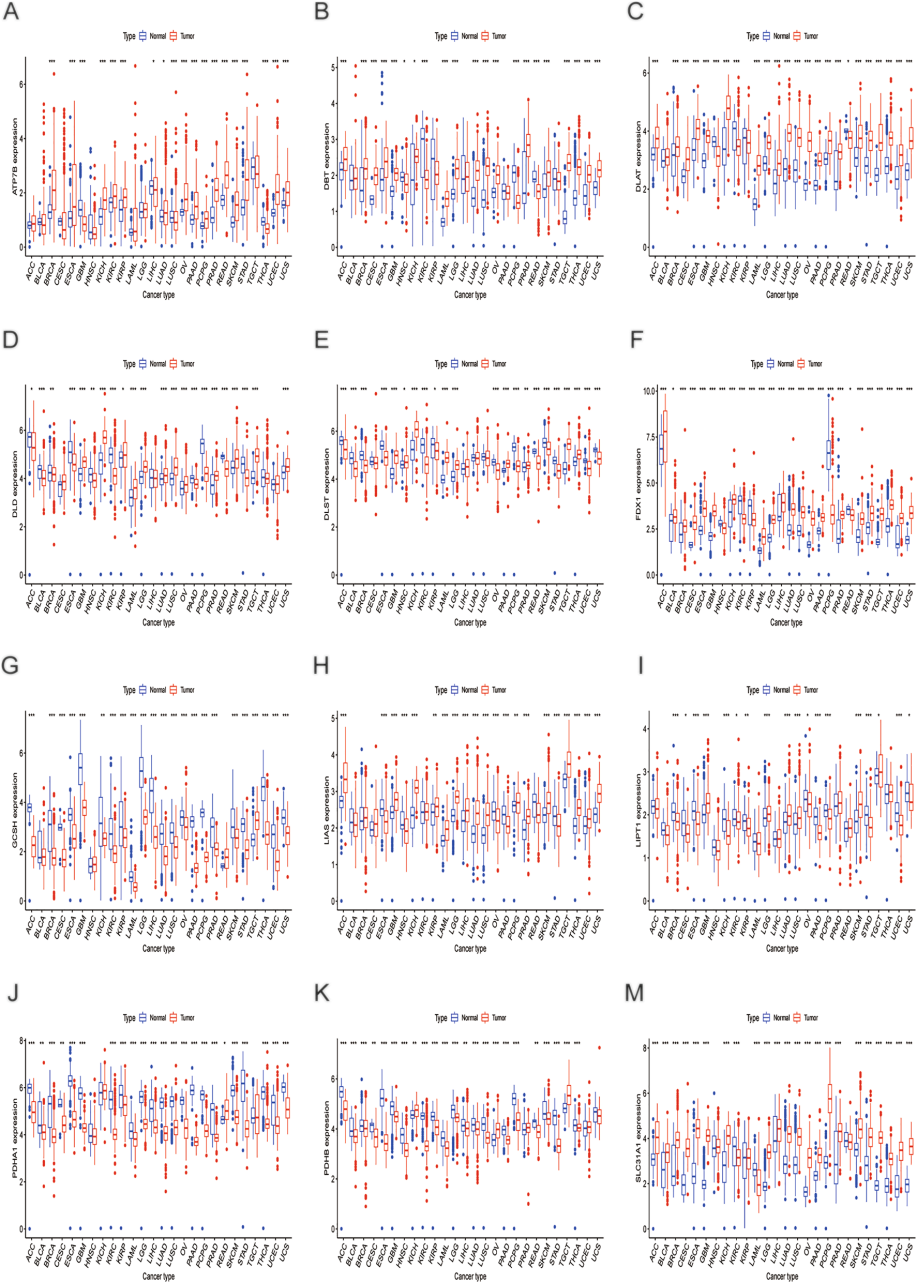
(**A**–**M**) Displays the cuproptosis-related genes expression levels in diverse tumor and normal tissues. The blue box plot represents normal tissues. The red box plot represents tumor tissues. *P < 0.05, **P < 0.01, and ***P < 0.001.

First, we undertook a comprehensive analysis of 26 different malignancies. The boxplots indicated that the gene level of FDX1 was dysregulated across all forms of cancer (Fig. 1). GCSH was lower expression in 24 cancers, except for BLCA, HNSC, READ, and TGCT. On the contrary, the expression of SLC31A1 was higher in 21 cancers, except LAML, KIRC, READ, HNSC, and KIRP. Supplementary Fig. S1. depicts the correlation analysis of the expression of CRGs in 26 cancers, most of which shared a positive correlation. However, ATP7B and GCSH, and FDX1 had a negative correlation. Compared to para-cancerous tissues, CRGs expression was dysregulated in numerous cancers, and CRGs may serve as oncogenes or tumor suppressors in these malignancies.

### Prognostic value of CRGs in cancer

**Figure 2:**
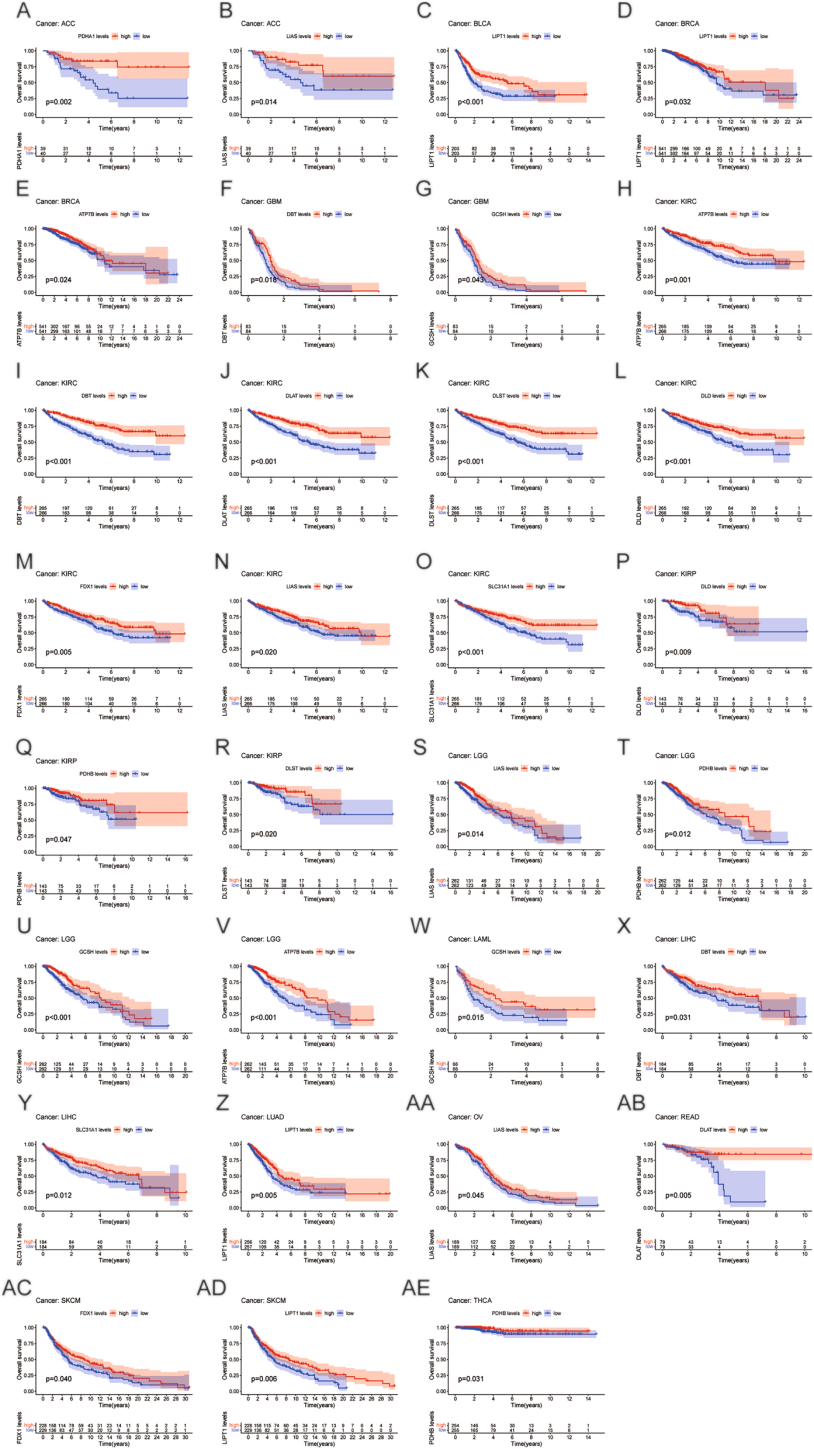
Survival analysis of cuproptosis-related genes positively associated with prognosis in various cancers.

We conducted prognostic value of CRGs expression across cancer. OS by KM analysis indicated that PDHA1 and LIAS were protective factors for patients with ACC, while FDX1 and SLC31A1 were risk factors. The analysis of the KM OS uncovered that FDX1 and SLC31A1 were risk factors for patients with LGG, while LIAS and PDHB were protective factors. Intriguingly, our results showed that DLAT, DBT, ATP7B, DLD, DLST, LIAS, FDX1, and SLC31A1 were protective factors for patients with KIRC. The results of KM survival analysis of CRGs in various cancers are presented in Fig. 2 and Supplementary Fig. S2.

The results of OS by Cox regression revealed that CRGs exhibit a notable correlation with patient prognosis in many forms of cancer. As shown in Supplementary Fig. S3, most CRGs are tightly connected to KIRC prognosis, except LIPT1 and PDHA1. Furthermore, PDHB and LIAS were protective factors for patients with KIRP and LGG, except for DBT and DLAT. However, OS by Cox regression showed that all CRGs showed no significant correlations with patient prognosis in GBM, KICH, OV, and STAD. These findings demonstrate a strong connection between CRG expression and patient survival with many forms of cancer.

### Analysis of the connection between CRGs and distinct immunological subtypes

**Figure 3:**
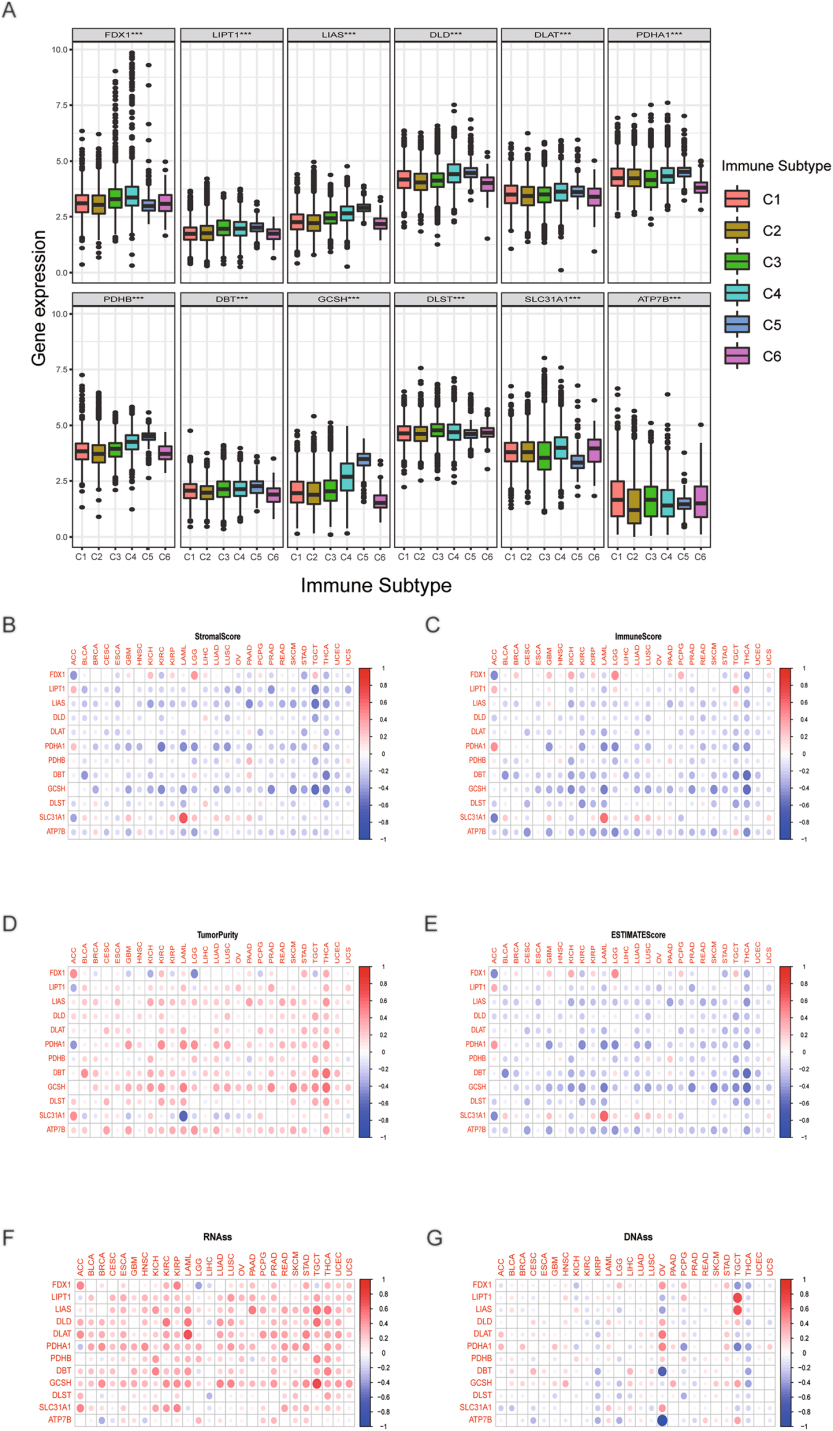
Analysis of the correlation between cuproptosis-related genes and distinct immunological subtypes and the tumor microenvironment, and tumor stemness scores. (**A**) Analysis of the correlation between cuproptosis-related genes and distinct immunological subtypes. (**B**–**E**) Correlation analysis between cuproptosis-related gene expression and stromal scores, immune scores, tumor purity, and ESTIMATE scores. (**F**) Correlation analysis between cuproptosis-related gene expression and stemness scores based on RNA expression. (**G**) Correlation analysis between cuproptosis-related gene expression and stemness scores based on DNA methylation.

Immune subtypes of malignancies vary, and each subtype of the immune system has distinct biological and clinical characteristics that influence tumor immune interactions^13^. Hence, we conducted an evaluation of the link between the expression of CRGs and six distinct immunological subtypes, including TGF-β dominant, immunologically quiet, lymphocyte-depleted, wound healing, inflammatory, and IFN-γ dominant. As shown in Fig. 3A, All CRGs exhibited significant association with various immune subgroups. For instance, the levels of FDX1 and DLST were greater in immune subtypes of C3 and C4 verse other immune categories. These findings suggest that CRGs may have a significant impact on tumor immunity.

### Correlation analysis of CRGs, the TME, and tumor stemness scores

A growing amount of studies have provided evidence to support that TME dictates cancer stem cell maintenance, which is crucial to cancer progression^14,15^. Therefore, we utilized the ESTIMATE method to determine the link between TME and CRGs expression in 26 distinct cancers. Our findings indicated that the CRGs were inversely linked with scores of ESTIMATE, stromal, and immunological across all malignancies (Fig. 3B,C,E). On the contrary, as shown in Fig. 3D,F, the TumorPurity, and RNA stemness score (RNAss) scores were positively correlated with CRGs. Furthermore, the DNA methylation-based stemness score (DNAss) scores was significantly related with OV, PCPG, and TCGT (Fig. 3G). Based on these findings, CRGs may significantly impact the tumor microenvironment.

### The relationship between CRGs and medication sensitively

Next, we analyzed the gene expression data of CRGs and IC_50_ using Pearson correlation analysis. Supplementary Fig. S4 demonstrated a positive connection between the expression levels of GCSH, FDX1, LIPT1, PDHA1 and the sensitivity to chelerythrine. Similarly, high LISA expressed resulted in more sensitively to palbociclib. On the contrary, DLD expression was inversely associated with the sensitively of oxaliplatin and dasatinib. These findings suggest that CRGs are response indicators in cancer treatment.

### Correlation analysis of CRGs and immune subtypes and TME in prostate cancer

**Figure 4:**
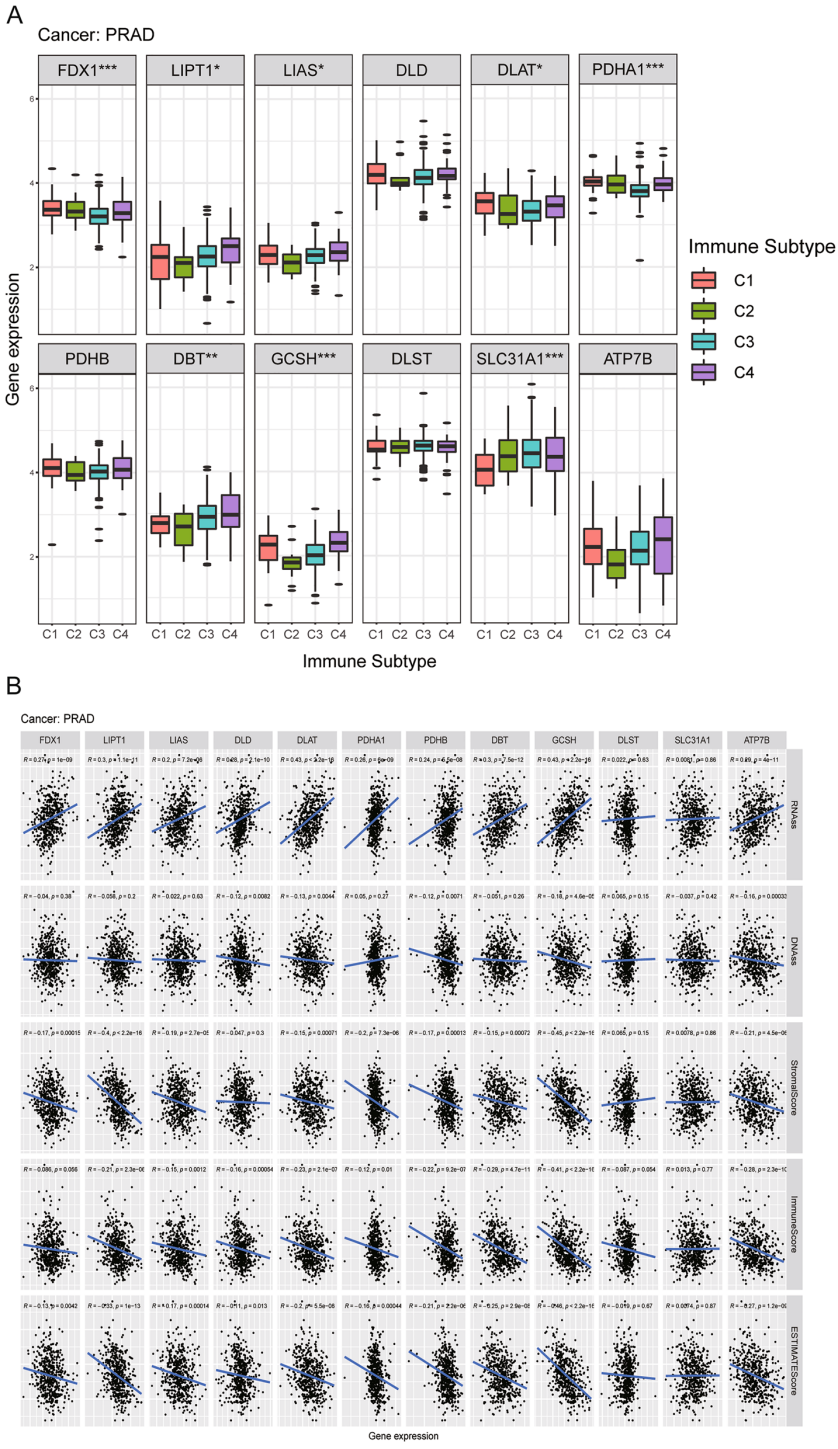
Correlation analysis of cuproptosis-related genes and immune subtypes and tumor stemness scores, and the tumor microenvironment in prostate cancer.

According to our previous analysis, CRGs expression is dysregulated in prostate cancer compared to other cancers, indicating that cuproptosis may potentially have a pivotal role in the pathogenesis of prostate cancer. In subsequent analysis, we analyzed the connection between CRGs and immune subtypes and TME. As shown in Fig. 4A, most CRGs, except for DLD, PDHB, DLST, and ATP7B, were tightly correlated with four immune subtypes, and most of them were highly expressed in several immunological subtypes. Our findings revealed all CRGs expression levels were positively connected with RNAss, but most of them were negatively with DNAss (Fig. 4B). Surprisingly, Most CRGs expression levels were negatively correlated with TME (the scores of ESTIMATE, stromal, and immune), indicating that the lower the level of CRGs, the greater the tumor purity. The above findings indicate that CRGs may significantly affect prostate cancer progression.

### Establishing and verification of a cuprotosis-related prognostic signature

Next, LASSO was used to create a prognostic signature. Supplementary Fig. S5A,B showed the LASSO profile plot and partial likelihood deviation, respectively. The survival data of prostate cancer patients from the TCGA were used to divide them into HR and LR groups. The OS rate was lower for patients in the HR group versus the LR group (*P* = 7.664e−03, Supplementary Fig. S5D). A time-dependent ROC analysis was applied to determine the specificity and sensitivity of risk signature. The AUC values for 36- and 60-month survival were 0.74 and 0.74, respectively (Supplementary Fig. S5C). Supplementary Fig. S5E,F display the risk score and survival status distributions, respectively, between HR and LR groups.

### Therapeutic response prediction

Next, we used anticancer drug sensitivity from the Genomics of Drug Sensitivity in Cancer database to compare the sensitivity of anti-tumor drugs between two groups. The results demonstrated that the sensitivity of the LR group to ABT.263, AZ628, ABT.888, A.443654, AZD.2281, axitinib, BI.2536, BIRB.0796, bleomycin, and BMS.509744 were higher than that of the HR group (Supplementary Fig. S6G–T). These findings suggest that our risk signature can facilitate the development of highly effective cancer treatments plans for prostate cancer patients.

### Analysis of single-cell transcriptomics

**Figure 5:**
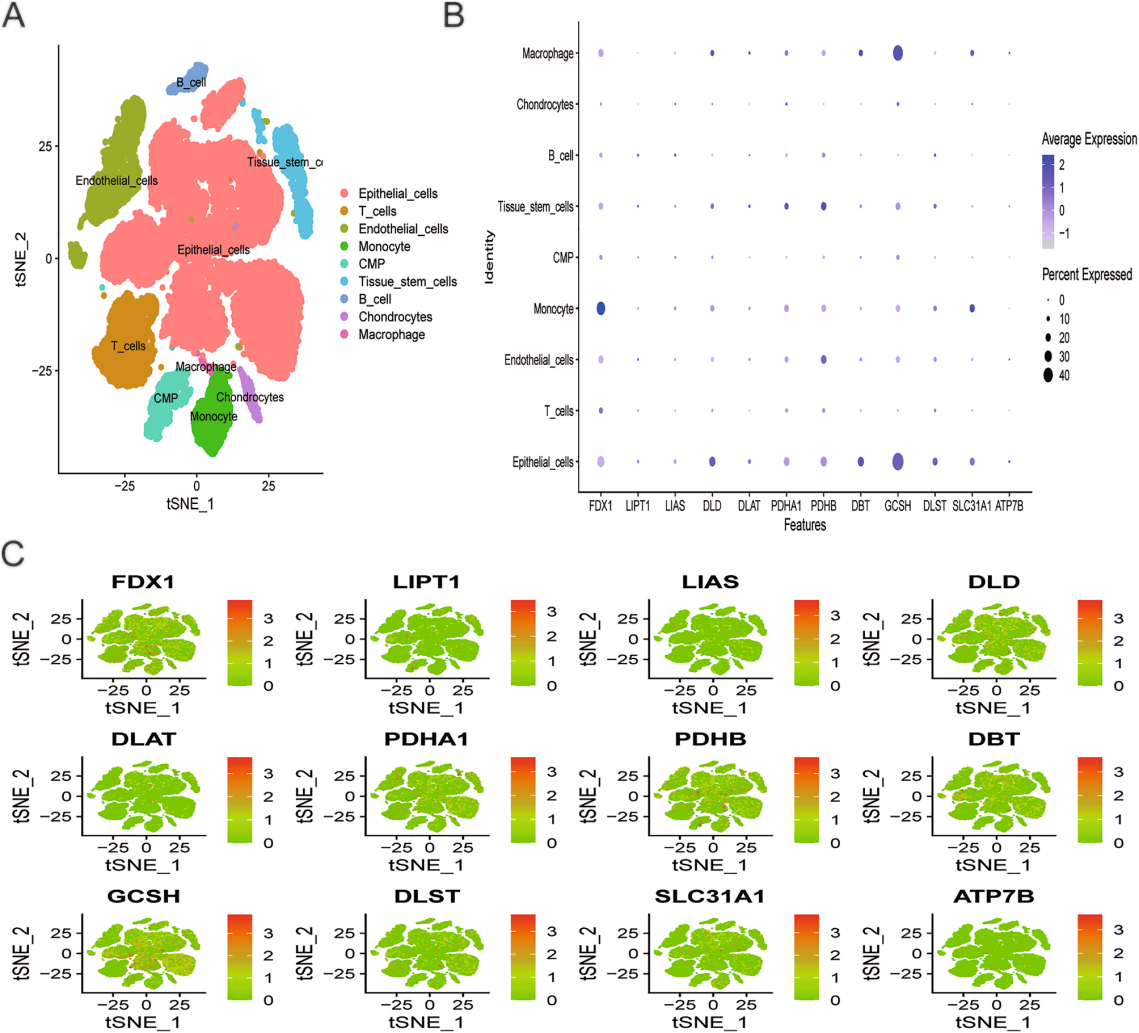
Single-cell RNA sequencing profiles of prostate cancer. (**A**) tSNE plots for 9 cell types. (**B**) The bubble plots show the expression levels of CRGs in all cell types. (**C**) The tSNE plots show the expression levels of CRGs in all cell types.

Sequencing depth in GSE141445 showed a positive correlation with mRNA count, but there was no significant correlation with mitochondrial gene sequence (Supplementary Fig. S7A,B), after implementing rigorous quality control measures. The principal component analysis effectively eliminated batch effects and identified the ideal number of principal elements (Supplementary Fig. S7C,D). Next, t-distributed stochastic neighbor embedding (tSNE) analysis was operated to downscale and visualize the data, resulting in the clustering of cells into 18 major clusters (Supplementary Fig. S7E). We have identified nine cell types, namely cells of epithelial, T, endothelial, monocyte, common myeloid progenitor, tissue stem, B, chondrocytes, and macrophage, based on their surface flag genes of various kinds of cells (Fig. 5A). Nine distinct cell types of CRG expression profiles were analyzed and demonstrated using a heatmap plot. Our results showed that FDX1, DLD, PDHA1, PDHB, GCSH, and SLC31A1 were predominantly expressed in cells of epithelial, monocyte, and macrophage (Fig. 5B). tSNE plots (Fig. 5C) were used to visualize the distribution of CRGs across various cell types.

### Elesclomol reduces the viability of prostate cancer cells by inhibiting FDX1 expression

**Figure 6:**
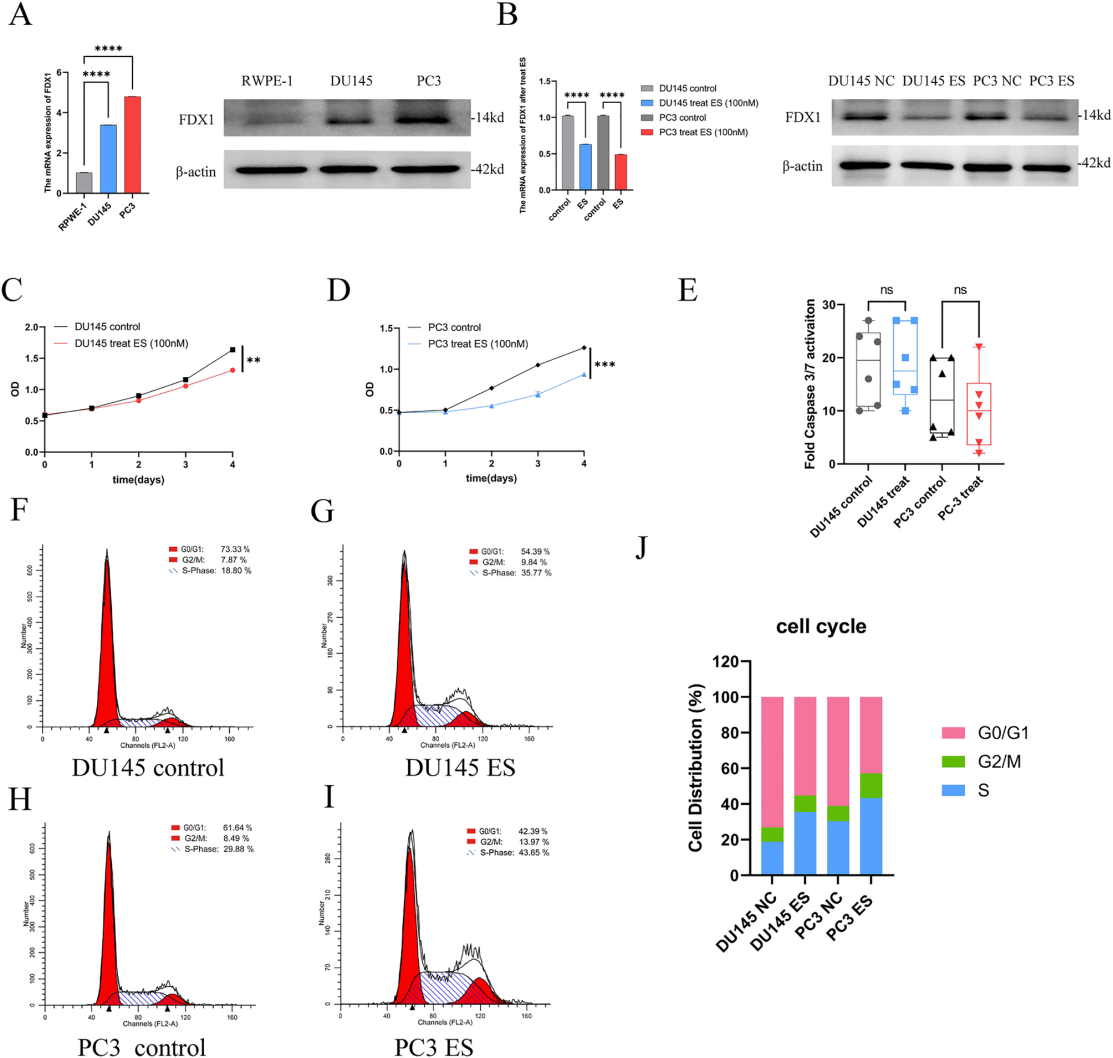
Elesclomol inhibits the viability of prostate cancer cells by downregulating FDX1. (**A**) The expression of FDX1 was investigated in RWPE-1 and prostate cancer cell lines by qPCR and western blot. (**B**) The expression of FDX1 in prostate cancer cell lines treated with elesclomol after 16 h. (**C**,**D**) The cell viability of prostate cancer cell lines after treatment with elesclomol. (**E**) The fold caspase 3/7 activity in prostate cancer cell lines after treatment with elesclomol for 16 h. (**F**–**J**) The cell cycle of prostate cancer cell lines after treatment with elesclomol.

**Figure 7:**
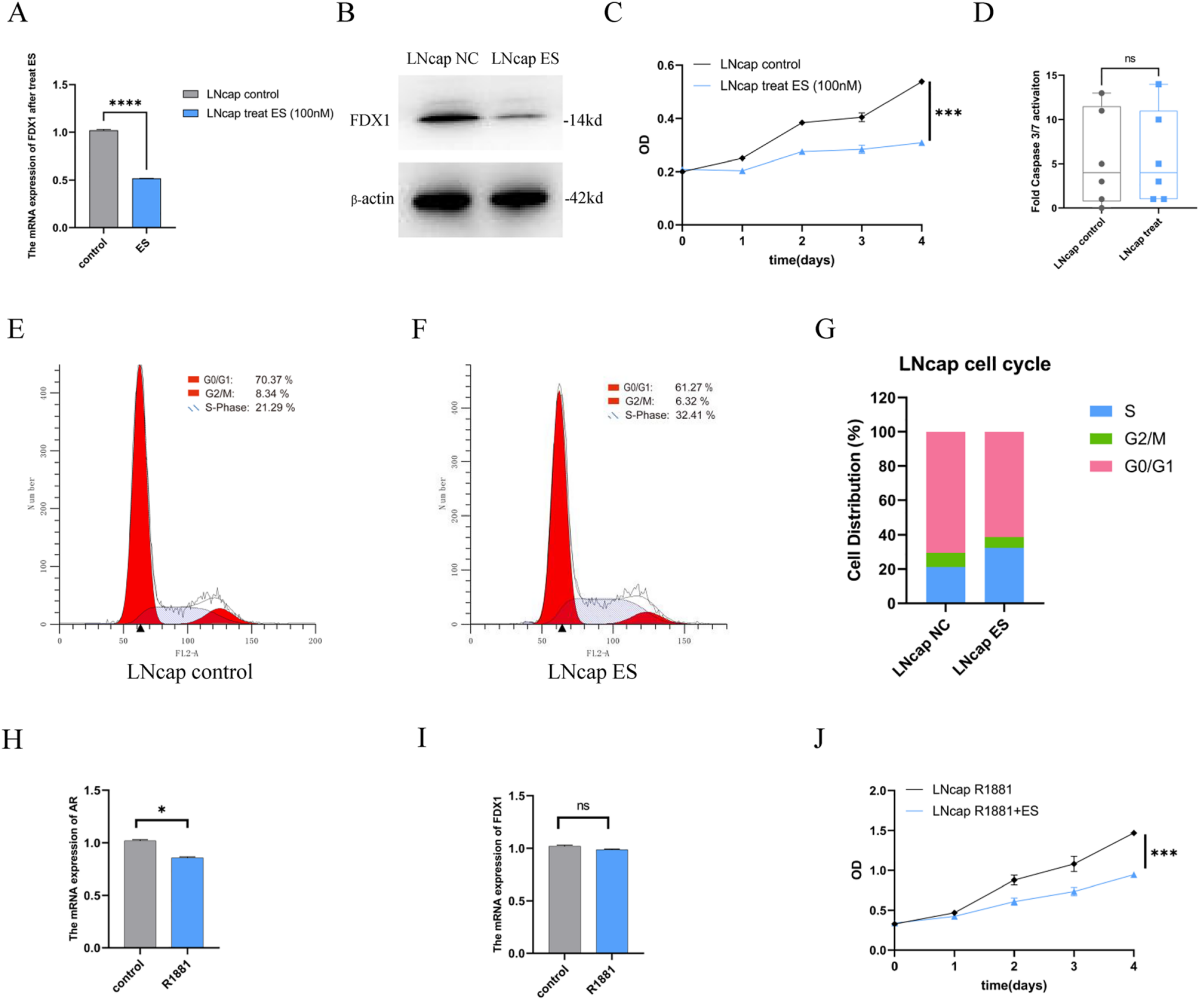
Elesclomol inhibits the viability of LNCaP cells by downregulating FDX1. (**A**,**B**) The expression of FDX1 in LNCaP cell lines treated with elesclomol after 16 h. (**C**) The cell viability of LNCaP cell lines after treatment with elesclomol. (**D**) The fold caspase 3/7 activity in prostate LNCaP cell lines after treatment with elesclomol for 16 h. (**E**–**G**) The cell cycle of LNCaP cell lines after treatment with elesclomol. (**H**,**I**) The mRNA expression levels of FDX1 and AR were examined in LNCaP cell lines following treatment with 0.1 nmol of R1881. (**J**) The cell viability of LNCaP cell lines was assessed following treatment with 0.1 nmol of R1881 alone or a combination of R1881 and elesclomol.

To confirm the findings of the above investigation, the level of FDX1 expression in prostate cancer cells was first examined. Figure 6A demonstrated that the levels of FDX1 expression were found to be considerably elevated in DU145 and PC3 cells compared to RWPE-1 cells. Based on prior research, it has been determined that ES specifically targets FDX1 and facilitates cell death that is dependent on copper^12^. Our results showed a striking decline in the proportion of living cells in ES treatment versus non-treated group (Fig. 6C,D). Meanwhile, the ES treatment group exhibited downregulation of FDX1 (Fig. 6B). Apoptosis did not contribute to copper-dependent cell death; next, we determined the effect of ES on caspase3/7 activity in prostate cancer cells. According to the data presented in Fig. 6E, there was no significant increase in caspase3/7 activity observed in ES treatment versus non-treated group. Additionally, we examined the effect of ES on the cell cycle. The percentage of during S phase cells distinctly enhanced from 18.80 to 35.7% and increased from 29.88 to 43.65%, respectively, after ES treatment in DU145 and PC3 cells (Fig. 6F–J). We also validated the role of ES in the androgen receptor-positive prostate cancer cell line-LNCaP. Our findings showed that ES suppressed cell proliferation and FDX1 expression while increasing the percentage of S phase cells without affecting caspase-3/7 activity (Fig. 7A–G). A hint from previous research indicated that the exposure of LNCaP cells to 0.1 nM of the synthetic androgen, R1881, led to a significant stimulation of cell growth^16^. In our study, we observed a noteworthy decrease in the expression levels of androgen receptor (AR) mRNA after exposing the LNCaP cells to 0.1 nM of R1881. Conversely, no significant alterations were observed in the levels of FDX1 (Fig. 7H,I). In addition, our findings revealed a significant decrease in LNCaP cell proliferation when treated with a combination of ES and R1881 compared to treatment with R1881 alone (Fig. 7J). Collectively, our findings indicate that the expression of FDX1 is suppressed by elesclomol, leading to a decrease in cellular viability via causing cell cycle arrest during S phase.

## Discussion

Cancer is a leading contributor to mortality and a significant impediment to quality of life globally, and there are currently no absolute cures for cancer^4^. The utilization of immune checkpoint inhibitors (ICIs) for management of malignancies has been reported, but the response to ICIs varies among patients^3^. Cuprotosis, is induced by elesclomol, a unique novel cell demise pathway involving many genes, binds lipoylated elements of the tricarboxylic acid (TCA) cycle directly, causing the death of human breast cancer cells^12^. However, there are few discoveries about CRGs and cancers. In this study, we analyzed CRGs across various malignancies. Finally, a validation experiment in prostate cancer cells was explored.

First, we investigated the expression of CRGs across cancers and discovered that all CRGs were expressed at a higher level in numerous malignancies. Moreover, KM and Cox regression survival analyses demonstrated that CRGs have predictive value in a variety of cancer types, revealing whether these genes were high-risk or low factors. Indeed, most of those prognostic CRGs have been observed to be linked to progression and prognosis of cancer patients. Prior findings showed that patients with ovarian cancer with high ATP7B expression were more likely to respond to cisplatin/carboplatin^17^. High DLST expression in individuals diagnosed with triple-negative breast cancer and neuroblastoma promotes cancer development by utilizing the TCA cycle and oxidative phosphorylation^18,19^. Inhibition of PDHA1 increases glycolysis and glucose consumption, which promotes esophageal squamous cell carcinoma and hepatocellular carcinoma growth^20,21^. Elesclomol specifically targets FDX1 in human BRCA and LUAD cells, decreases iron cluster biosynthesis mediated by FDX1 and induced copper-dependent cell demise^12^. In vivo, silencing DLD suppressed melanoma development and reduced TCA cycle-related metabolites^22^. PM2.5-induced up-regulation of DLAT and dysregulation of glycolytic metabolism was found to facilitate non-small-cell lung cancer carcinogenesis^23^. LIPT1 was found to reduce cell migration in BLCA cell lines and to be associated with better patient survival^24^.

The immune subtypes of malignancies vary, and each has unique biological features that impact anticancer treatment to a certain degree^13^. Our research examined the relationships between CRGs and immunological subtypes. The results indicated a strong association between all CRGs and different immunological subgroups, such as the expression of FDX1 were found to be increased in immunological subtypes C3 and C4. Supporting that, C3 had the best outcome, while C2 and C1 had worse outcomes, even though they had a huge amount of immune cells. Also, C4 and C6 have a greater degree of heterogeneous immunological features and displayed the least favorable survival outcomes^13^. These results suggested that CRGs could provide additional predictive indicators for immunological subtypes and could act as prognostic marker tumor immune response. TME, which includes tumor purity and scores of immune, stromal, and ESTIMATE, has been found to exert a significant influence on tumor development, and response to therapeutic interventions^14^. Our results suggested that CRGs were correlated with scores of ESTIMATE, stromal, and immune, revealing the influences of varied stromal and immune cell infiltration and tumor purity. Tumor stemness is one of the most critical sources of chemotherapeutic resistance, which could be increased by TME to enhance those efforts^25^. Our results indicate a strong correlation between CRGs and stemness scores. These findings illustrate that the stem cell characteristics of cancer patients can be predicted according to the expression of CRGs.

Mounting evidence indicated that novel advancements in cancer treatment had been established, including targeting the ubiquitin–proteasome pathway^26^, tumor suppressor gene^26^, and RNA interference^27^. However, drug resistance was also a significant challenge in preclinical and clinical therapies. We investigated the relationship between the IC_50_ of over 70 anticancer medicines and the expression of CRGs. According to our findings, several drug sensitivities were shown to be closely correlated with CRGs. For instance, the sensitivity to chelerythrine was positively connected with the expression level of GCSH, FDX1, LIPT1, and PDHA1. Chelerythrine, an inhibitor of protein kinase C, has demonstrated promising anticancer effects in several malignancies^28–31^. These data demonstrates that that modulating the expression of CRGs is another potentially useful strategy for improving the therapeutic value of anticancer drugs.

Next, we analyzed the role of CRGs in prostate cancer due to their expression almost dysregulation than other cancers. Our results showed that CRGs were discovered to be closely correlated with the immunological subtype, tumor stemness scores, and TME in prostate cancer, which suggests that modulating of CRGs expression may be a potential approach for enhancing the effectiveness of immune therapy. Validation of the levels of expression using datasets from single-cell sequencing also indicated that some CRGs were predominantly present in immunological cell and tissues stem cell. Not surprisingly, previous research has demonstrated that the expression of SLC31A1 and PD-L1 and CD16+ (NK cells), CD8+, CD4+ (T cells) was downregulated by Dextran-Catechin treatment^32^. Furthermore, the expression level of ATP7B is closely connected with drug resistance and cancer stemness^33^. Inhibition of DLST decreased immunosuppressive marker expression and function in bone marrow-derived myeloid of the ovarian cancer mouse model^34^. We constructed a CRGs prognostic signature in prostate cancer using LASSO analysis and revealed OS was poorer for patients in HR compared to LR group, indicating that CRGs may be a promising predictor for prostate cancer patients. In terms of other cancers, high expression of ATP7B was found in GBM and was found to be connected with lower OS times of patients with GBM^35^. LIPT1 inhibited cell migration in bladder cancer cell lines and contributed to favorable survival in bladder cancer patients^24^. Recent research revealed that the utilization of cabazitaxel and docetaxel as primary therapeutic interventions for individuals with metastatic castration-resistant prostate cancer^36^. Therefore, we evaluated the chemotherapeutic sensitivity of HR and LR patients. Our findings suggested that the LR group was more sensitive to ABT.263, AZ628, ABT.888, A.443654, AZD.2281, axitinib, BI.2536, BIRB.0796, bleomycin, and BMS.509744 than the HR group was, which could facilitate the manufacturing of highly effective or therapeutically valuable medications.

Finally, the effect of the cuproptosis-key gene FDX1 on prostate cancer cells were investigated through an in vitro experiment. These findings indicated that prostate cancer cell lines exhibit a substantially greater level of FDX1. Furthermore, elesclomol suppressed cell survival without requiring caspase 3 and 7 activations and reduced FDX1 expression, consistent with prior findings^12^. Additionally, elesclomol reduced the growth of prostate cancer cells via arresting the S phase. These findings indicate that elesclomol inhibits FDX1 expression and decreases prostate cancer cell survival without requiring caspases 3 and 7 activations.

However, this research has some flaws. Firstly, the data was obtained mainly from public databases. Consequently, in vivo validation of our findings is necessary. Secondly, although we proved that elesclomol decreased the cell survival of prostate cancer cells by targeting FDX1, the underlying regulatory mechanisms requires more investigation.

In conclusion, we conducted the most comprehensive investigation of the relationships between CRGs and tumor characteristics, such as expression differences, surviving outcomes, immunological subtypes, stemness scores, the TME, and treatment sensitivity, and validated the results in prostate cancer. Notably, elesclomol decreased cell survival in prostate cancer cells by targeting FDX1. These findings may shed fresh light on the mechanism of CRGs across malignancies and pave the way for identifying potential treatment targets.

### Supplementary Information

Supplementary Information.

## Data Availability

The datasets are available from TCGA database (http://cancergemome.nih.gov/) and GTEx database (http://gtexportal.org/home/), and GEO database (https://www.ncbi.nlm.nih.gov/geo/). The study’s original findings are provided within the paper and Supplementary Material. For further inquiries, please contact the corresponding authors.

## Abbreviations

CRGs: Cuproptosis-related genes
TME: Tumor microenvironment
PD-1: Programmed cell death-1
PD-L1: Programmed cell death-ligand 1
CTLA4: Cytotoxic T lymphocyte-associated antigen-4
β2MG: Beta 2-microglobulin
HLA: Human leukocyte antigen
KM: Kaplan–Meier
IC_50_: Half maximal inhibitory concentration
TCGA: The Cancer Genome Atlas
GTEx: Genotype-Tissue Expression
SLC31A1: Solute carrier family 31 member 1
DLAT: Dihydrolipoamide *S*-acetyltransferase
ATP7B: ATPase copper transporting beta
DLD: Dihydrolipoamide dehydrogenase
DBT: Dihydrolipoamide branched chain transacylase E2
DLST: Dihydrolipoamide *S*-succinyltransferase
GCSH: Glycine cleavage system protein H
LIAS: Lipoic acid synthetase
FDX1: Ferredoxin 1
LIPT1: Lipoyltransferase 1
PDHB: Pyruvate dehydrogenase E1 subunit beta
PDHA1: Pyruvate dehydrogenase E1 subunit alpha 1
OS: Overall survival
TGF-β: Transforming growth factor beta
IFN-γ: Interferon gamma
HR: High risk
LR: Low risk
GEO: Gene Expression Omnibus
qRT-PCR: Real-time quantitative polymerase chain reaction
ES: Elesclomol
RNAss: RNA stemness score
DNAss: DNA stemness score
LASSO: Least absolute shrinkage and selection operator
GDSC: Genomics of Drug Sensitivity in Cancer
PCA: Principal components analysis
tSNE: T-distributed stochastic neighbor embedding
ICIs: Immune checkpoint inhibitors
TCA: Tricarboxylic acid
