# Supplementary material for: Comprehensiveness cuproptosis related genes study for prognosis and medication sensitiveness across cancers, and validation in prostate cancer
Source: Sci Rep. 2024 Apr 26;14:9570. doi: 10.1038/s41598-024-57303-8 (PMC11053037; doi:10.1038/s41598-024-57303-8)

**Comprehensiveness cuproptosis-related genes study for prognosis and medication sensitiveness across cancers, and validation in prostate cancer**

Longfei Yang^1,2,4,5^, Yifan Tang^2,3,4,5^, Yuwei Zhang^1,2,4,5^, Yang Wang^2,3,4,5^, Peng Jiang^2,3,4,5^, Fengping Liu^2,3,4,5*^, and Ninghan Feng^1,2,3,4,5*^

**Supplementary Material**


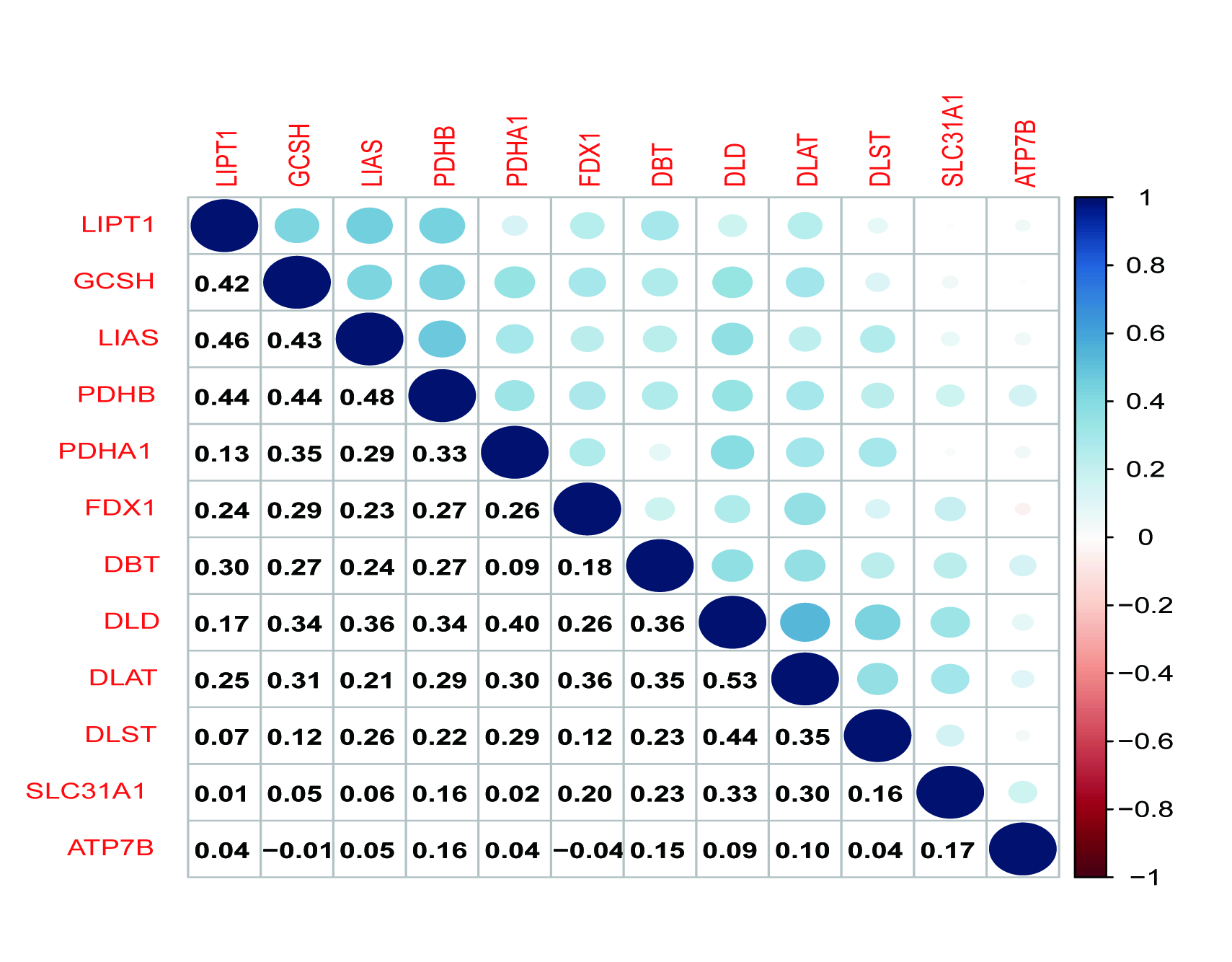


S1. Correlation analysis of cuproptosis-related genes. Blue and red dots, respectively, represent positive and negative correlations.


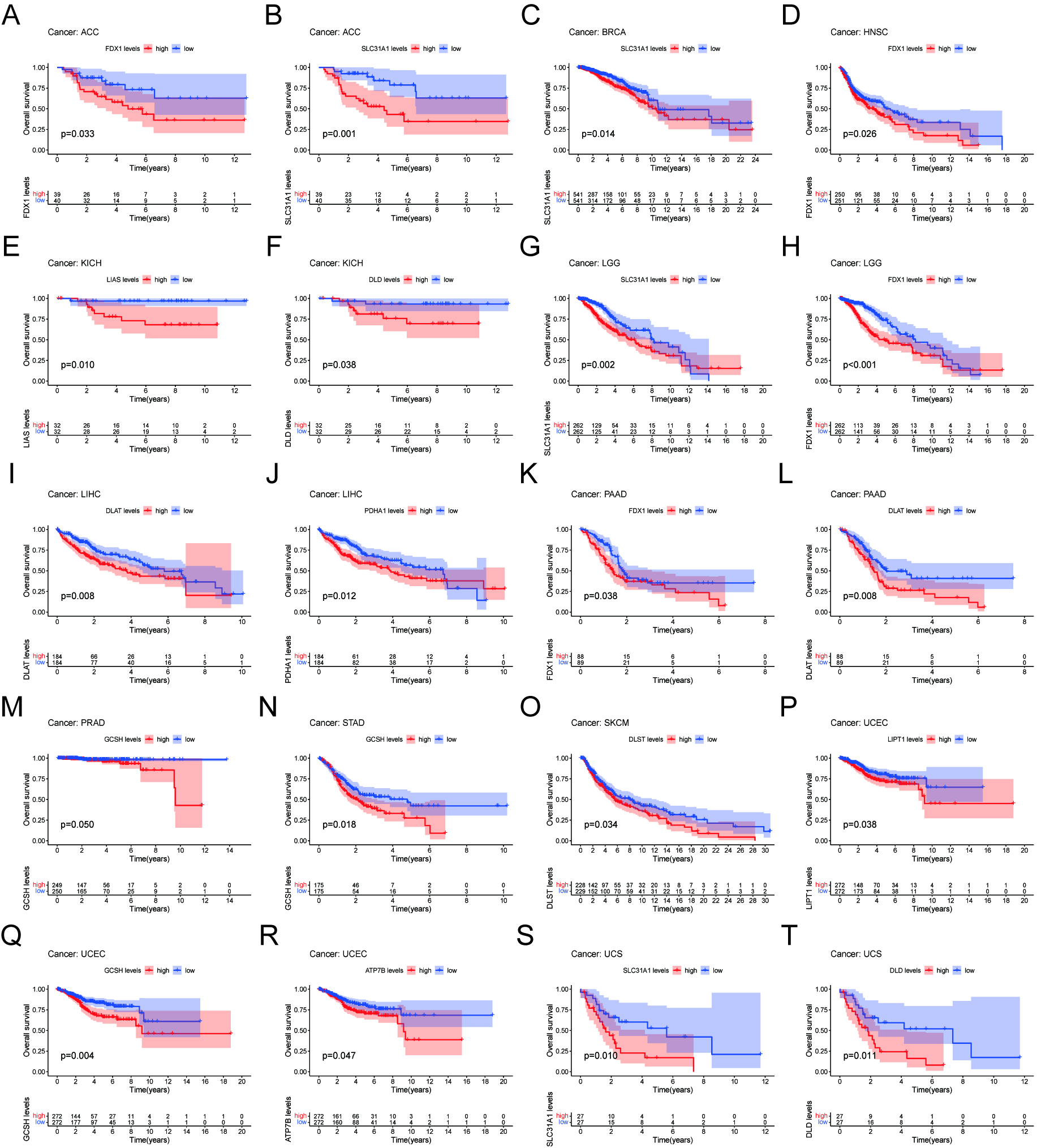


S2. Survival analysis of cuproptosis-related genes negatively associated with prognosis in various cancers.


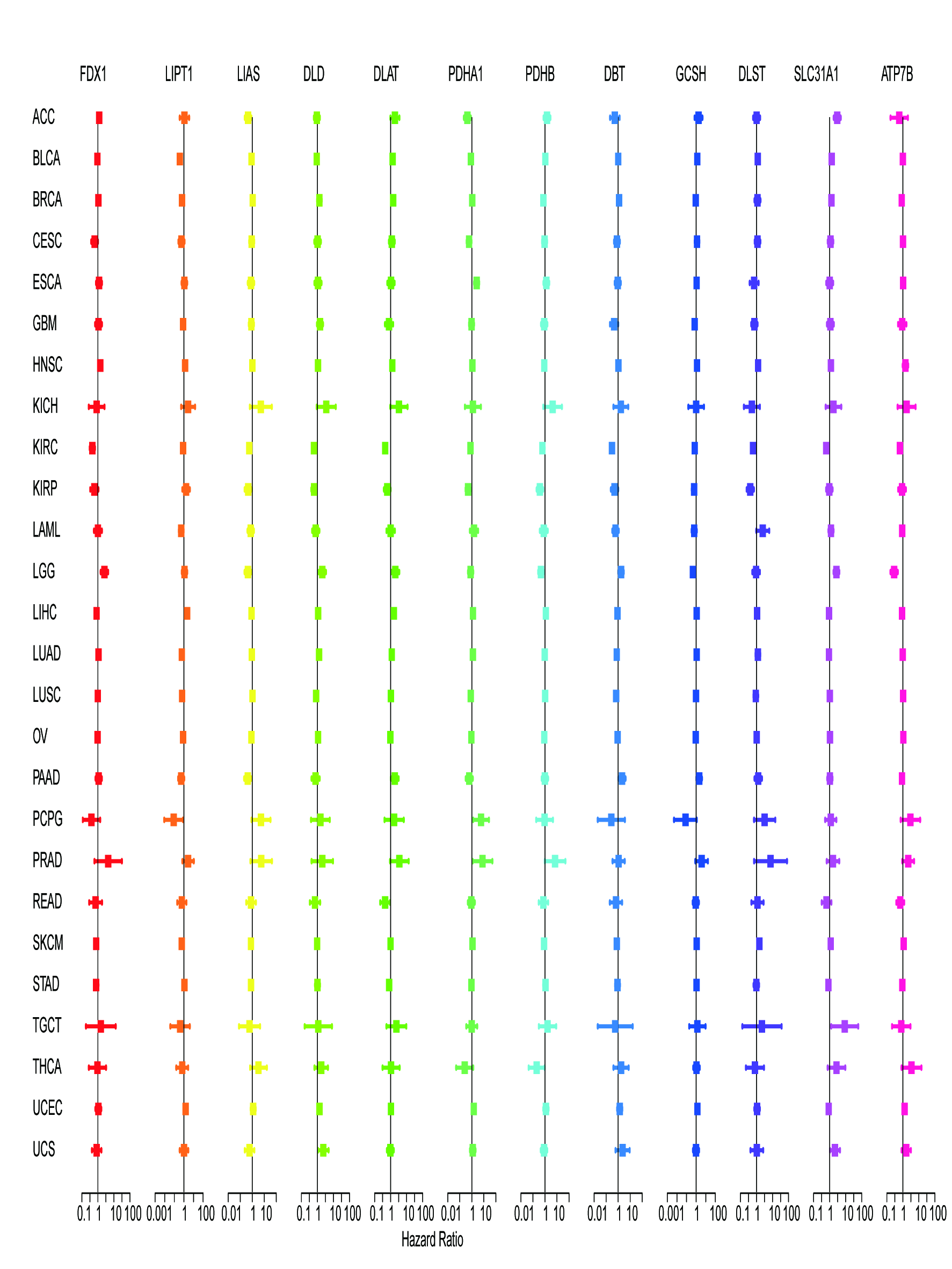


S3. Cox regression analysis reveals a correlation between cuproptosis-related genes expression and overall survival in distinct forms of cancer. A hazard ratio > 1 represents high risk, and a hazard ratio < 1 represents low risk.


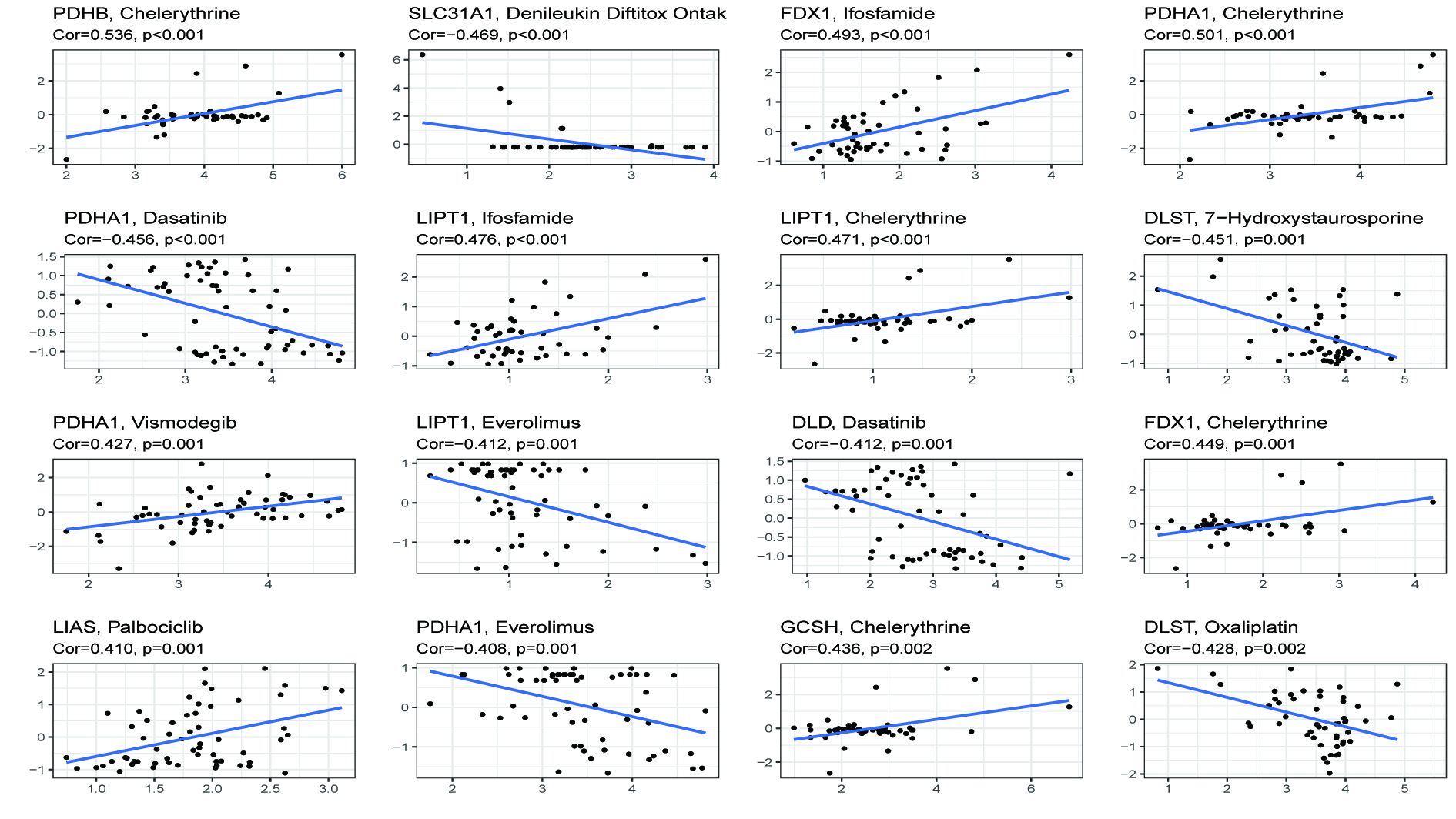


S4. The relationship between cuproptosis-related genes and drug sensitivity.


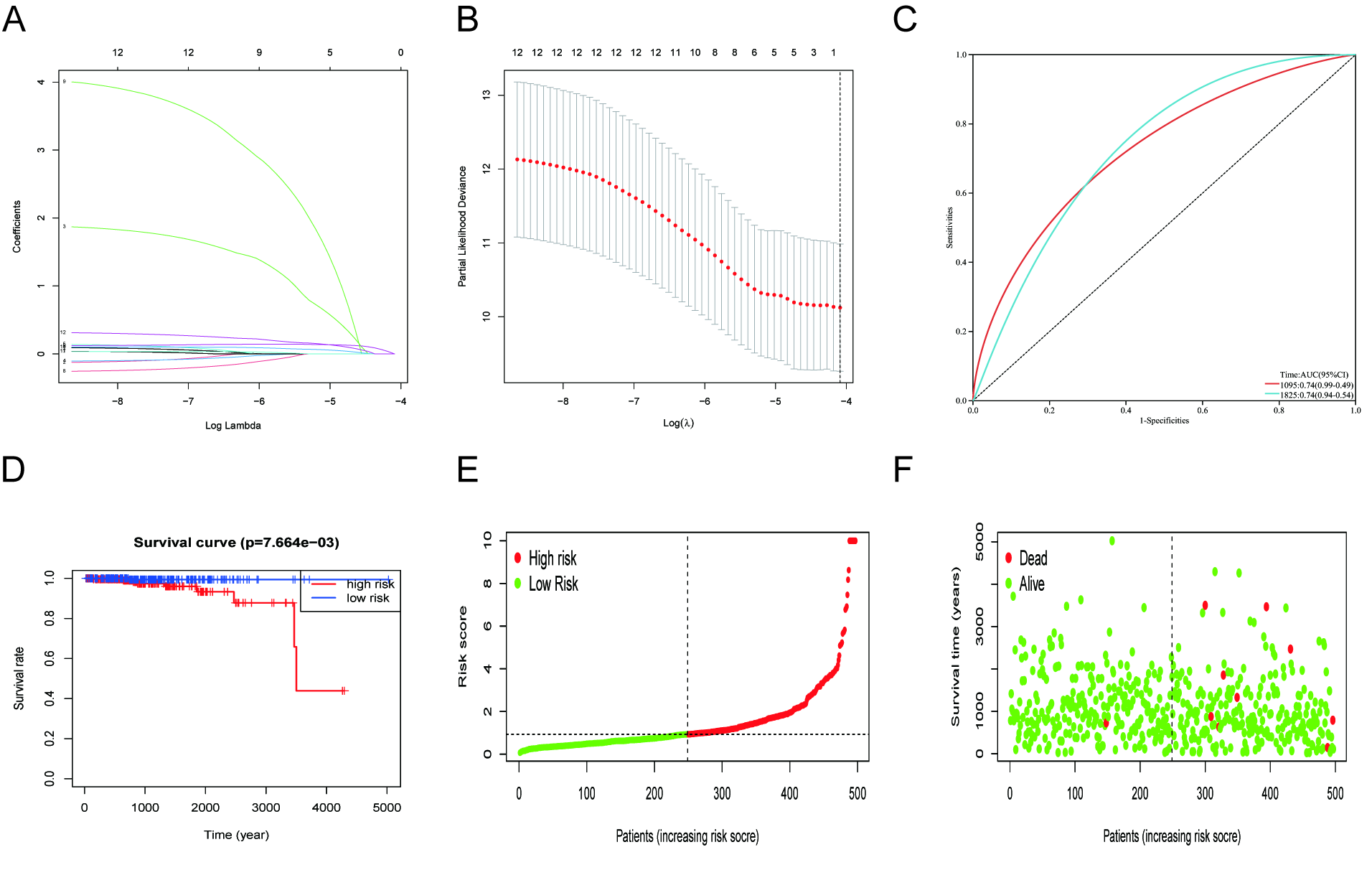


**S5**. Construction and validation of a cuprotosis-related prognostic signature. (A) Least absolute shrinkage and selection operator (LASSO) regression of the OS-related genes. (B) Cross-validation for tuning the parameter selection in the LASSO regression. (C) The ROC curve of measuring the predictive value of the risk model. (D) KM curves for the OS of patients in the high- and low-risk groups. (E) Risk score distributions of each patient. (F) Survival status distribution of each patient.


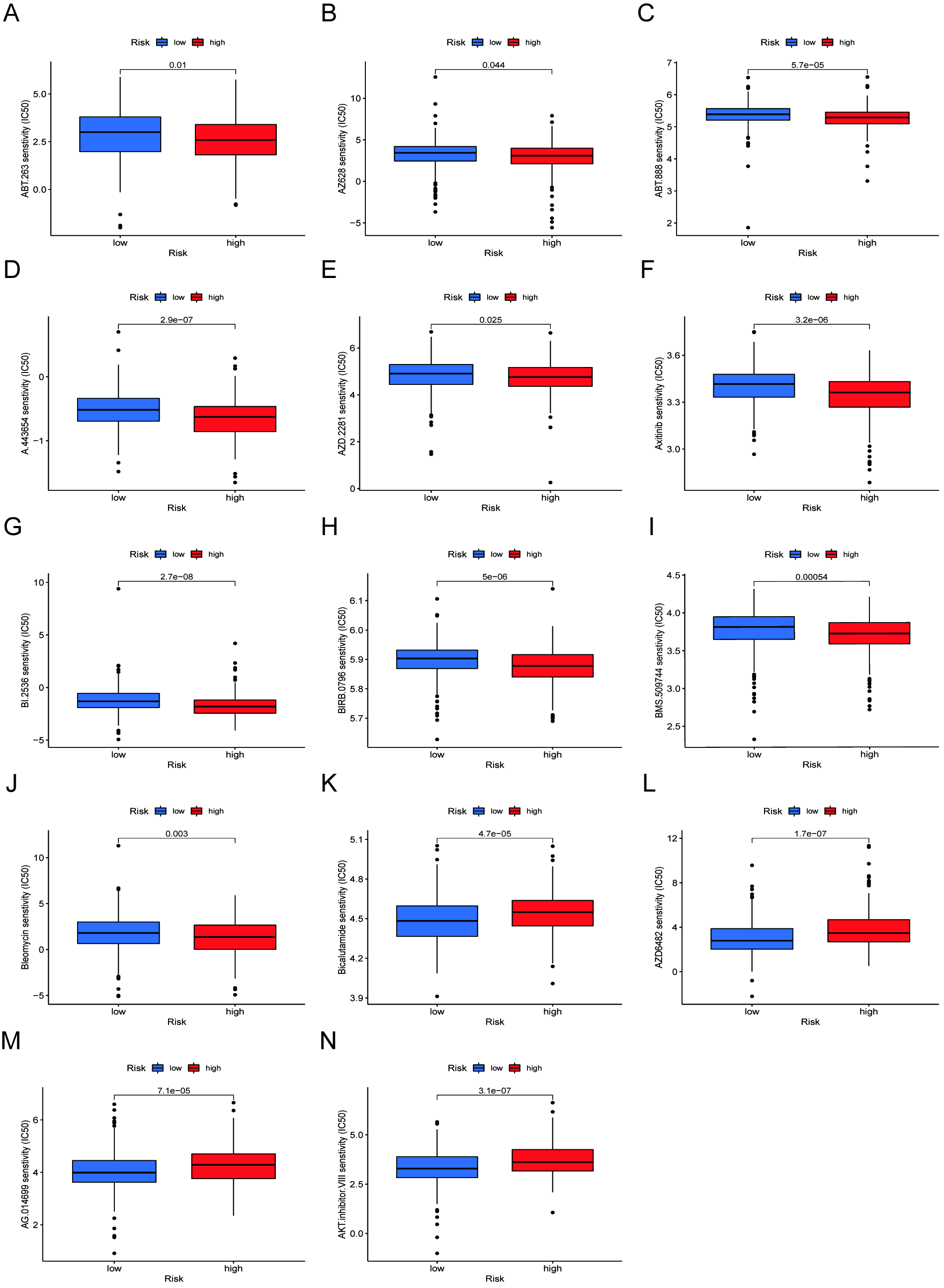


**S6**. The correlation between the risk score and therapeutic response prediction.


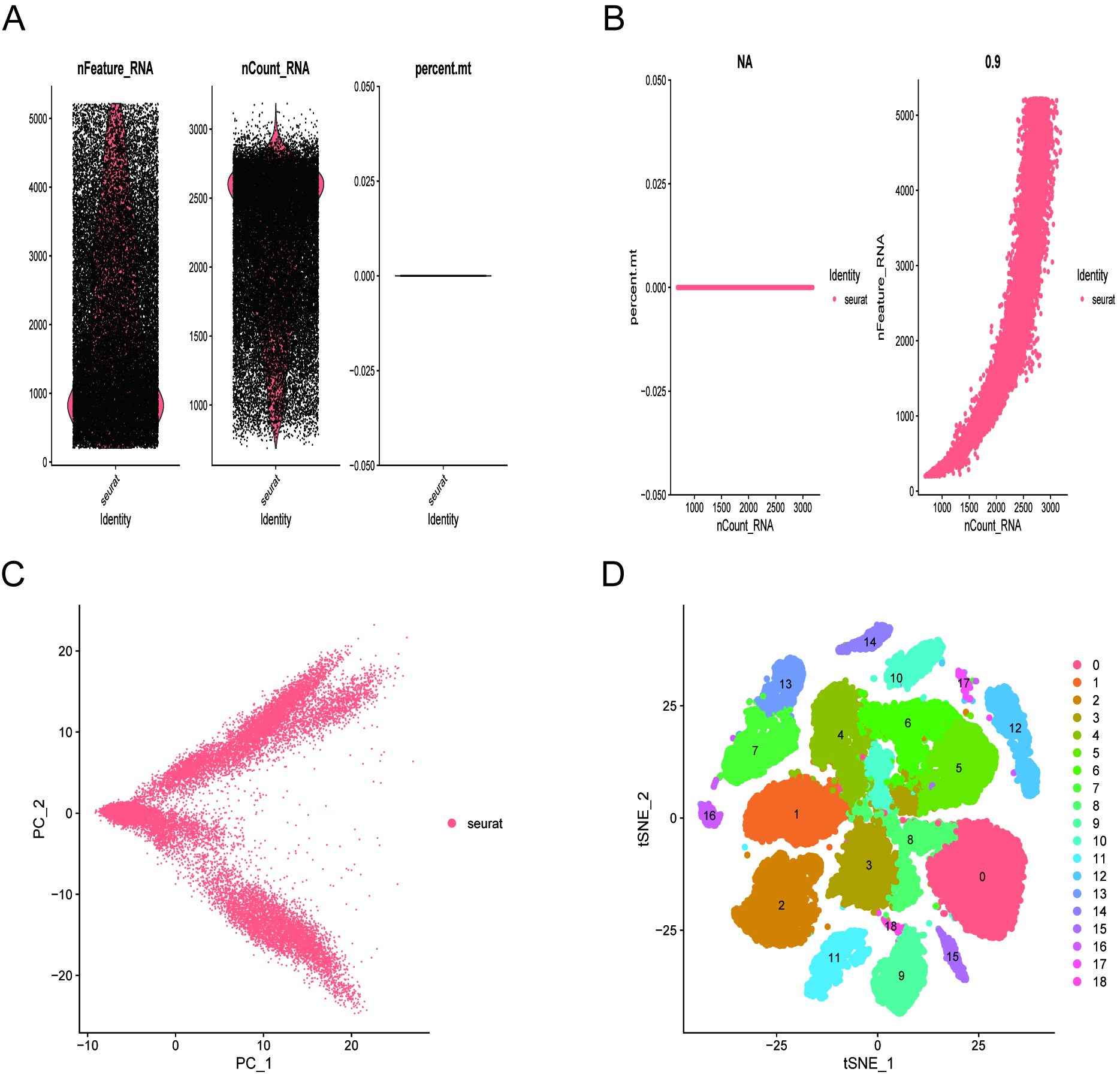


**S7**. Analysis of PRAD tissues by single-cell RNA sequencing. (A, B) After quality control, each sequenced cell is filtered with violin plots that show its RNA feature number (nFeature RNA), absolute UMI count (nCount RNA), and mitochondrial content (percent. mt), as well as their relationship. (C)PCA of single-cell data from seven different tissues. (D) The UMAP dimensionality reduction algorithm clustered cells into 19 types, with each color representing the annotated phenotype of each cluster.

**Table S1:** RT-qPCR primer sequences

| Gene name | Sequence (5′-3′) |
| --- | --- |
| FDX1 | Forward: TTCAACCTGTCACCTCATCTTTG |
|  | Reverse: TGCCAGATCGAGCATGTCATT |
| β-actin | Forward: GACGTGGACATCCGCAAAG |
|  | Reverse: CTGGAAGGTGGACAGCGAGG |


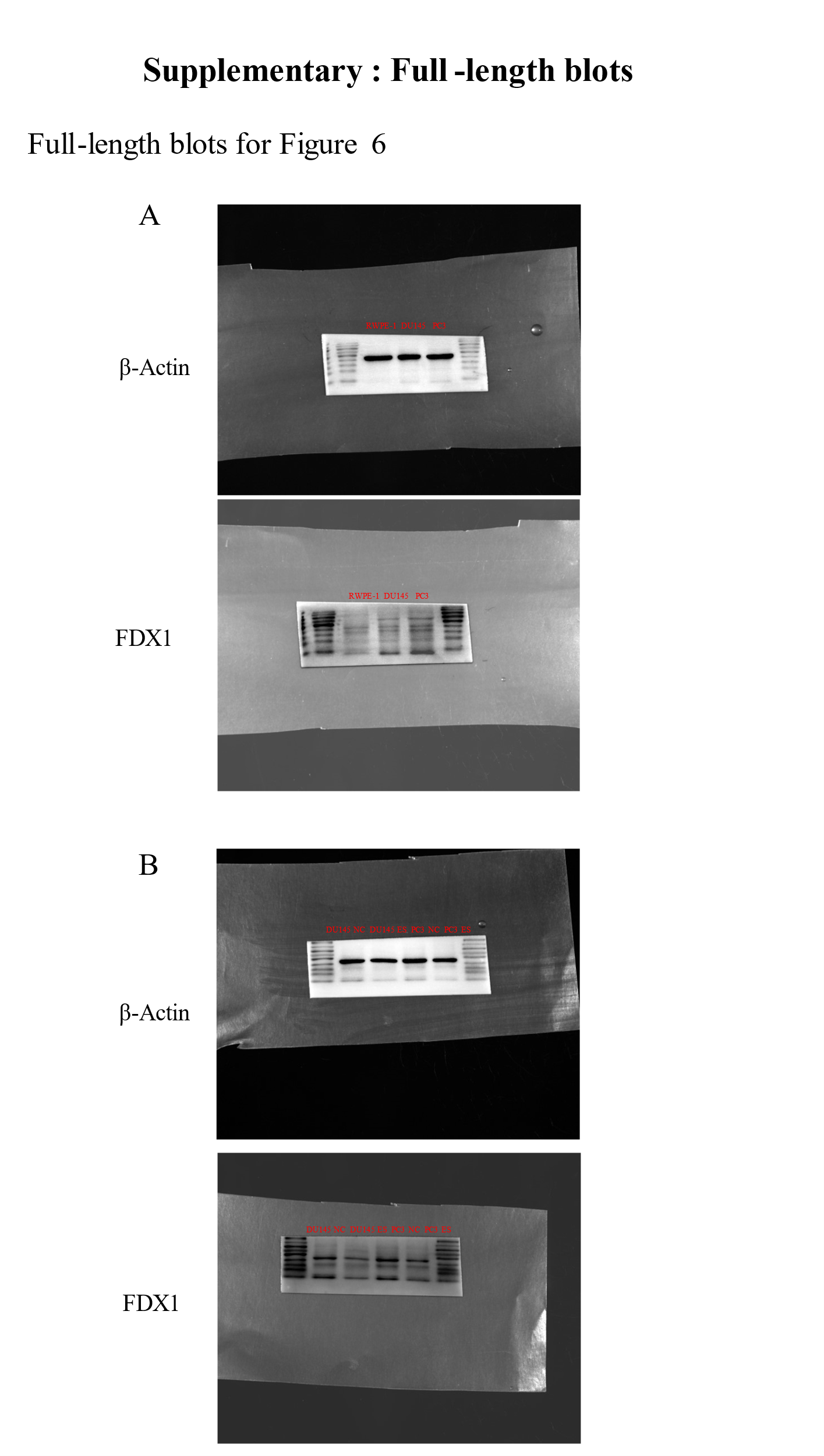


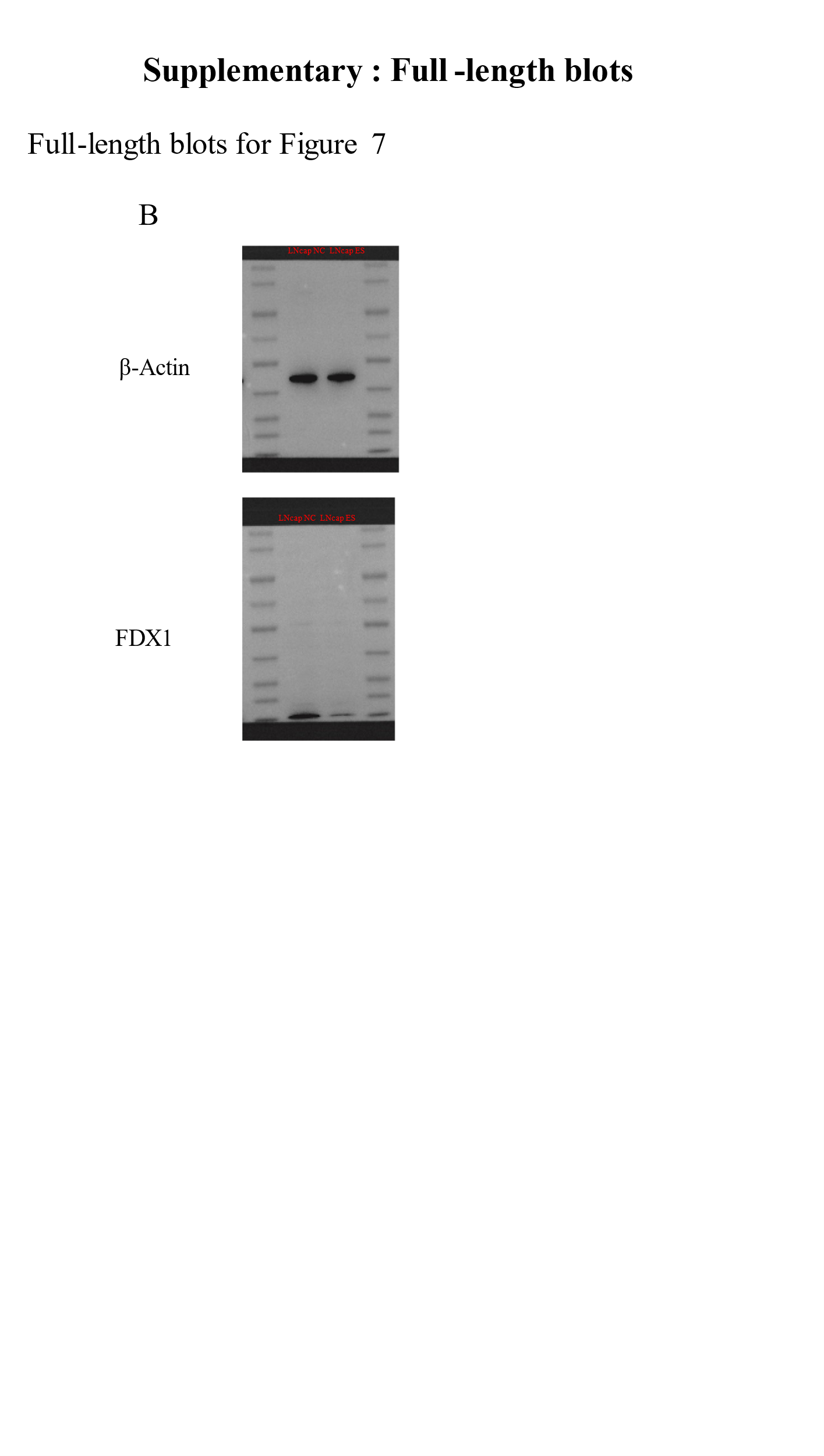

Supplement: Supplementary file 1 — Supplementary Information. [file 41598_2024_57303_MOESM1_ESM.docx]
